# Famous Poisoners in Fiction

**Published:** 1893-09-23

**Authors:** 


### FAMOUS POISONERS IN FICTION.

### V.—THE POISON MAID.

THIS charming tale* is of the character of an allegory. It tells of a sublime truth. Love, the all conqueror, prevails; love, the vision of youth, of happiness, triumphs, and the short story gives to the reader new hope, new courage. It is, however, a regular *fin de siècle* story, no plot, no sustained interest; only just a perfect character sketch, lightly, charmingly written, telling of how the noble can turn revenge to love, of how the poisoned body can be subdued by a soul full of the beauty of purity and holiness.

Magnificent is Mithridata's self-abnegation to the bitter end. "If it must be so I choose the scaffold," is her noble answer to the king's command. The story can be told in a very few words. It much resembles Hawthorne's delightful "Rapacini's Daughter;" the American author has worked out the idea more fully but not so artistically.

Mithridata, the daughter of the magician Locusto, was educated by her father in an extraordinary manner. Enormous serpents infested her cradle, licking her face and twining around her limbs. Her father fed her "with spoonfuls of the honeyed froth that gathers under the tongue of asps, and when she craved a more nutritious diet she partook, at first in infinitesimal doses, but in ever-increasing quantities, of arsenic, strychnine, opium, and prussic acid."

And yet she grows up to be, to again quote Dr. Richard Garnett's own words, "without a peer for beauty, sense, and goodness." Then one day he tells her his terrible secret, tells her, "Thy kiss would be fatal to anyone not fortified by a course of antidotes," and afterwards gives his reason for her strange up-rearing. She was to be a means to an end, that end, *revenge.* Her father owed a grudge to the king of the land. "His father slew my father," he declares; "wherefore it is meet that I should slay that ancestor's son's son. Go," he bids her, "to the great city. Thou art beautiful as the day; he is young and handsome and amorous; he will infallibly fall in love with thee."

Mithridata refuses to fulfil her horrible mission, for, as she wisely argues, "If I do not love him I am no wise minded to suffer him to caress me. If I do love him, I am as little minded to be the cause of his death." Her father turns her out of doors for this act of disobedience, grumbling, as fathers are apt to grumble, at the disappointing of his most cherished plans. "They will not so much as commit a murder to please us," he angrily opines. Homeless, disinherited, Mithridata arrives at the great city, and sadly pathetic are the descriptions of this fair woman, who now denies herself the poison she has learnt to love, and who dares not bestow a kind look on those around her. Yet she could not succeed in keeping the men from loving her, or rather her beauty, and fate brought to her door the very young prince whose death her father desired.

Mithridata, fainting one day at the sight of blood shed on her account, found herself, on recovering from her swoon, in the arms of a beautiful youth, who was sucking death, *i.e.*, kisses from her lips, with extraordinary relish. It was, of course, the prince, and equally, of course, Mithridata loved him. But the charming tale has no tragic ending. It concludes as so many love stories do, and all love stories ought, with a wedding and marriage bells. The old king had been too clever for the magician, he had fed his son on antidotes. So the wedding peal rang out gaily over the heads of the happy lovers, and Locusto is bidden to the marriage feast to learn there "that the kiss of love is the remedy for every poison."

Dr. Garnett's beautiful story, the last in the book, entitled "Twilight of the Gods and Other Tales" is well worth reading if but for the exquisite English in which it is written, and all men, but especially doctors and nurses, who see so much of the shadowed side of life, and know by experience how often remedies are ineffectual to cure, will rejoice in the ending, with its cheery words of hope. Aye, love, the undying, shall conquer every foe, vanquish every poison of the soul, shall heal every heartache, and cure all sorrows, when it shall reign victorious in the Heaven of Heavens.

The idea of a maid prepared by poison for the destruction of an enemy is not the offspring of any nineteenth-century author's brain. It has been a legend of the land of Ind from time immemorial, a whisper from the ages of the past which the credulous accept as history, but the unbelieving practical scientist condemns as impossible. It is most likely one of the half mythical fables which, as they were repeated from generation to generation, came to be clothed in the garment of fact. Unable to prove or disprove the story as it stood, men were fain to believe it to be the actual truth. Reference is made to this time-honoured legend in the preface to the Hindu play, "Mudra Rakshasa," translated from the Sanscrit by Professor Wilson, and to which our attention was kindly drawn by Dr. Garnett himself. Rakshasa, we here read, "prepared by magic art a poisoned maid for the destruction of that prince (Chandragupta), but Kantilya detected the fraud, and diverting it to Parvatésa caused his death."

But if the idea of the poisoned maid be untenable by common sense and by medical science the idea of resisting poisons by a course of antidotes is not so wholly fictitious. The very name of our poison maid, "Mithridata," shows that the author is well acquainted with the tradition about Mithridates the Great, King of the Bosporus, the renowned enemy of the Roman generals, Lucullus and Pompey. He, surrounded by foes from his childhood, took the precaution of wearing armour against the blows of sword or spear, and of strengthening by antidotes his system against the more insidious attacks of poison. So effective were his medicines that when, in old age, he, defeated and dishonoured, sought by its means to rid himself of the life he now hated, he found his efforts vain; the plan which had proved successful against his enemies now proved equally so against himself. He died at last by a stroke of a sword administered by his own hand, his daughters, Mithridatis, or Mithridata, and Nyssa, with the reckless disregard for life so common amongst the ancients, taking poison at the same time. This idea of "*A bon chat bon rat*" is a favourite one n many

* "The Twilight of the Gods and other Tales." By RICHARD GARNETT, author of "A Life of Carlyle," "Idylls and Epigrams," &c.

English and French novels, for, after all, there is nothing new under the sun, and the same means of evil, the same plans of escape, the same old tales of love, of jealousy, of revenge, were told in the days of eld, just as books were then also written, only laboriously and carefully on rolled papyrus instead of being the work of the machine, and possessing a bright showy cover, if of little instrinsic worth. And the cycles of time move ever onward, onward, and the little span allotted to man passeth quickly by, yea, even as a dream of the night, and as a tale that is told, until on the last page we read the word—blotted by the tears of mourners—"Finis." And "the end of all things draweth nigh."

